# Supplementary material for: Taxonomic and systematic revisions to the North American Nimravidae (Mammalia, Carnivora)
Source: PeerJ. 2016 Feb 9;4:e1658. doi: 10.7717/peerj.1658 (PMC4756750; doi:10.7717/peerj.1658)
Supplement: Supplemental Information 1 [file peerj-04-1658-s001.docx]

Specimens examined during the course of this study. Organization of this section is presented by institution, and taxonomy (Family) of specimens examined.

**South Dakota School of Mines and Technology, Museum of Geology (SDSM)**

Nimravidae

SDSM: 55280, 521, 15013, 73866, 5776, 4053, 4081, 2417, 5946, 28153, 3666, 2687, 68878, 6939, 536, 2641, 2661, 28139, 69054, 293, 2564, 2882, 3670, 56140, 5311, 2528, 2416, 2662, 69261, 28145, 15012, 54247, 2544, 2865, 2663, 348, CP 5095 (field number).

**University of Nebraska State Museum (UNSM)**

Nimravidae

UNSM: 48454, 26607, 1072, 279-51, 26402, 27007, 25505, 1070, 20-14-8-34 SP,

2-4-8-33NP, 25492, 25506, 25512, 1068, 322-51, 2033-53, 1888-38, 3003-63 A, 25750, 1022-38, 25748, 25524, 25768, 25771, 2509-59.

Amphicyonidae

UNSM: 25785, 26173.

**American Museum of Natural History (AMNH), Frick Collection (FAM)**

Nimravidae

AMNH: 6941, 98770, 38980, 102375, 62022, 1407, 38951, 6938, 102156, 62124, 1403, 1399, 6931, 6933, 6930, 455, 12882, 104631, 105390, 21638, 55562, 11067, 6936, 6937, 8777, 5335, 38805, 39101, 9764, 32668, 38982, 38981, DICK 33-1370 (field number), LUSK 309-2729 (field number), LUSK 0143-3052 (field number).

FAM: 98189, 69377, 98769, 62075, 102387, 62025, 102155, 62026, 62019, 62042, 62151, 104823, 62074, 62070, 62073, 125655, 69403, 125651, 69370, 125657, 62077, 125658, 62125, 69420, 125662, 125675, 69427, 62091, 62084, 69424, 125660, 62007.

Amphicyonidae

AMNH: 70801, 68243, 55597, 6851, 6852, 6853.

FAM: 68241, 49239, 83386, 76207, 50329.

Miacidae

AMNH: 15176, 143785, 129284, 19000, 19198, 11495, 15644.

Viverravidae

AMNH: 2830, 17030, 14750, 4790, 15180, 15174, 48696, 15609, 55484, 15992, 3372, 16540, 15995.

**Yale Peabody Museum (YPM),**

**Yale Peabody Museum Princeton University (YPM PU)**

Nimravidae

YPM: 56858, 10053, 10045, 1044, 10517, 10046, 14388, 10519, 14385, 10049, 10052, 10050, 10051.

YPM PU: 12953 A, 11372, 13635, 12515, 11256, 16271, 11569, 11079, 10502, 10972, 12536, 12558, 12577, 13798, 13628, 12750, 13582, 12751, 12698, 11379, 12573, 13587, 13795, 14999, 13625, 21452, 12957, 10540, 10515, 10741, 12749, 12542, 12590,

12750 A, 13136, 12972, 10647.

Amphicyonidae

YPM: 10066, 56883, 24612, 10064, 12714.

YPM PU: 11421, 13792.

Miacidae

YPM: 11839, 10071, 13040, 13070, 14217, 12845, 11861, 13041, 14218.

**United States National Museum (USNM)**

Nimravidae

USNM: 18219, 15889, 18214, 12820, 16812, 15890, 18187, 18193, 18191, 18189, 18181, 15891, 18203, 18185, 15912, 15951, 18190, 18183, 15926, 18192, 18194, 18182, 15902, 100, 15925, 362744, 25146, 99, 3957, VHVP 735 (field number).

Miacidae

USNM: 5920, 214706, 362796, 362789.

Viverravidae

USNM: 362834, 362820, 22802, 19470.

**Badlands National Park (BADL)**

Nimravidae

BADL: 10593, 59734, 30677, 4893, 59490.
