# Supplementary material for: Taxonomic and systematic revisions to the North American Nimravidae (Mammalia, Carnivora)
Source: PeerJ. 2016 Feb 9;4:e1658. doi: 10.7717/peerj.1658 (PMC4756750; doi:10.7717/peerj.1658)
Supplement: Supplemental Information 2 [file peerj-04-1658-s002.docx]

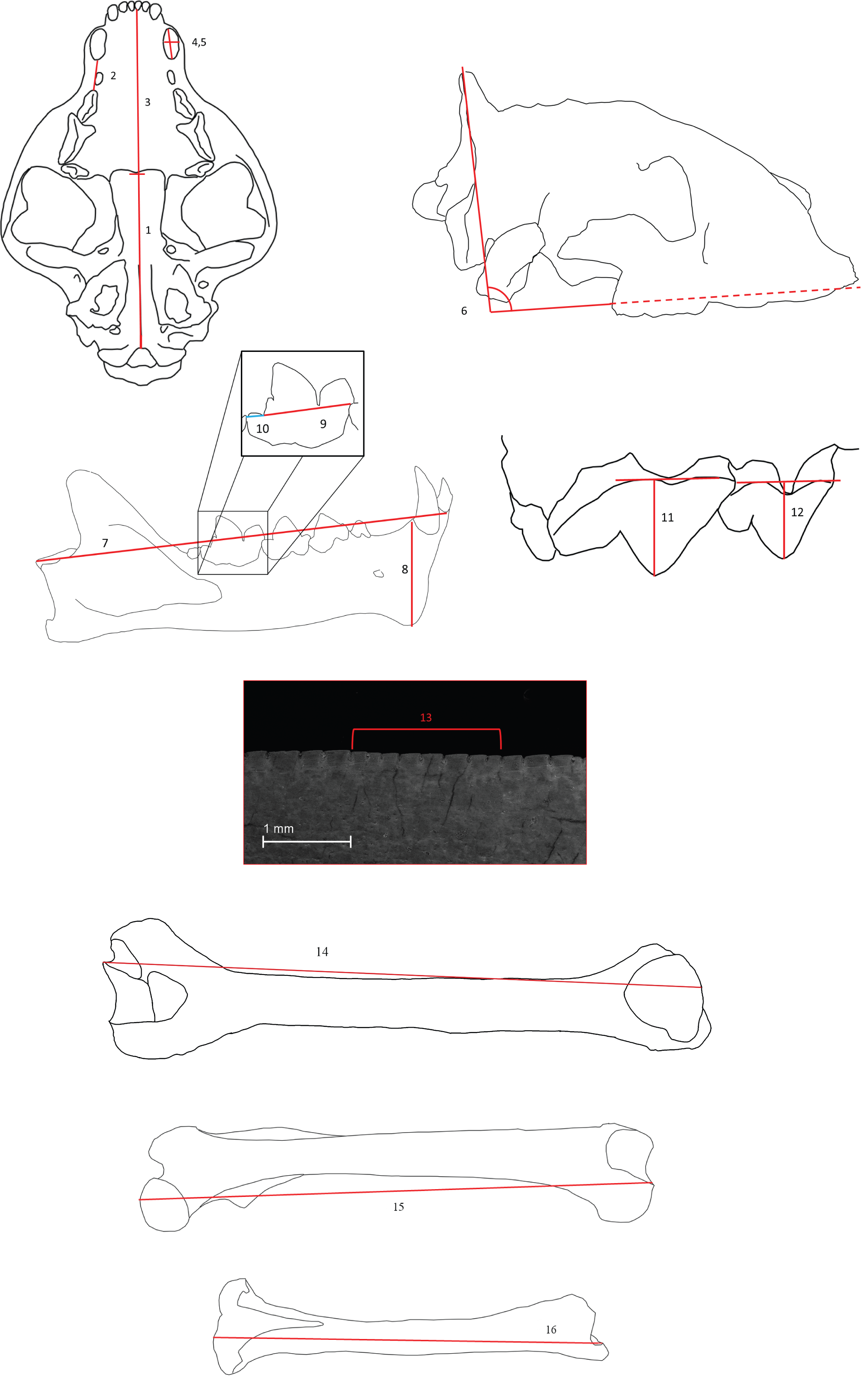


**Figure S2.** Measurements taken on specimens to quantify differences between species. (1): basilar length, prosthion to basion; (2): post canine diastema length, disregarding possible P1-2s; (3): palate length, prosthion to staphylion; (4): canine length, at enamel-dentine boundary; (5): canine width, at enamel-dentine boundary; (6):angle of lambdoid crest, measured as intersection of palate and prominent trajectory of crest; (7): mandible length, from articular process to anterior-dorsal most position of mandibular symphysis; (8): genial flange height, measured from directly behind c1 to the extremal tip of the flange; (9): trigonid length of m1; (10): m1 length; (11): P4 height, measured from base of cingulum to apex of tooth; (12): P3 height, measured from base of cingulum to apex of tooth; (13): serration density, measured over an average of 5 mm; (14): humerus length, measured to extremal points of articulation;(15): femur length, measured to extremal points of articulation; (16): tibia length, measured to extremal points of articulation.
