# Supplementary material for: Taxonomic and systematic revisions to the North American Nimravidae (Mammalia, Carnivora)
Source: PeerJ. 2016 Feb 9;4:e1658. doi: 10.7717/peerj.1658 (PMC4756750; doi:10.7717/peerj.1658)
Supplement: Supplemental Information 5 [file peerj-04-1658-s005.docx]

**R Code Utilized in Multivariate Analyses**

**Dinictis**

Data Imputation

Norm package

# removed taxa assignment column from Dinictis2.0t to create Dinictis2.0nt

Dm<-data.matrix(Dinictis2.0nt)

s<-prelim.norm(Dm)

thetahat <- em.norm(s) #find the MLE for a starting value

rngseed(1234567) #set random number generator seed

theta <- da.norm(s,thetahat,steps=100,showits=TRUE) # take 100 steps

ximp<-imp.norm(s,theta,Dm) #impute missing data under the MLE

Multivariate Normality

ICS package

mvnorm.kur.test(ximp)#checks for multivariate normality, essential for the following analyses

Cluster Analysis

Dinicdist<-dist(ximp,method= "euclidean") #creates distance matrix for cluster analysis

Dinicclust<-hclust(Dinicdist,method= "average") #UPGMA clustering

plot(Dinicclust)

#added taxa assignment column, as determined by cluster analysis

DFA

Caret, Mass packages

set.seed(3456)

trainIndex<-createDataPartition(ximp_t1$Taxon,p=0.5,list=FALSE) # ximp_t1$Taxon uses Taxon column to maintain proportional makeup of taxa in training/testing sets, p=0.5 performs 50/50 split in sets

data_train<-ximp_t1[trainIndex,]

data_test<-ximp_t1[-trainIndex,]

model<-lda(Taxon~.,data_train, prior=c(0.5,0.5)) #prior sets equal probability of a specimen being in either group to account for sample size differences

predictions<-predict(model, data_test[,-1]) # does not include taxon assignment in prediction

confusionMatrix(predictions$class, data_test$Taxon) #generates summary table and correct classification percentage

**Pogonodon**

Data Imputation

Norm package

# removed taxa assignment column from Pogoac to create Pogoacnt

Pm<-data.matrix(Pogoacnt) # data frame does not include taxa assignment column

s<-prelim.norm(Pm)

thetahat <- em.norm(s) #find the MLE for a starting value

rngseed(1234567) #set random number generator seed

theta <- da.norm(s,thetahat,steps=100,showits=TRUE) # take 100 steps

Pogoimp<-imp.norm(s,theta,Pm) #impute missing data under the MLE

Multivariate Normality

ICS package

mvnorm.kur.test(Pogoimp)#checks for multivariate normality, essential for the following analyses

Cluster Analysis

Pogodist<-dist(Pogoimp,method= "euclidean") #creates distance matrix for cluster analysis

Pogoclust<-hclust(Pogodist,method= "average") #UPGMA clustering

plot(Pogoclust)

#reinserted taxa assignment column for 2 groups, as supported by cluster analysis

DFA

Mass package

set.seed(3456)

model<-lda(Taxon~.,Pogoimpt2, prior=c(0.5,0.5),CV=TRUE) #prior sets equal probability of a specimen being in either group to account for sample size differences, and utilizes jackknife cross-validation due to small sample size (according to caret package documentation classes ≤ 3 may not show up in both training and testing sets with createDataPartition command)

ct <- table(Pogoimpt2$Taxon, model$class)
diag(prop.table(ct, 1))
# total percent correct
sum(diag(prop.table(ct)))

**Nimravus**

Data Imputation

Norm package

Nm<-data.matrix(Nimravus3.2nt) # data frame does not include taxa assignment column

s<-prelim.norm(Nm)

thetahat <- em.norm(s) #find the MLE for a starting value

rngseed(1234567) #set random number generator seed

Nimp<-imp.norm(s,thetahat,Nm) #impute missing data under the MLE

Multivariate Normality

ICS package

mvnorm.kur.test(Nimp)#checks for multivariate normality, essential for the following analyses

Cluster Analysis

Nimdist<-dist(Nimp,method= "euclidean") #creates distance matrix for cluster analysis

Nimclust<-hclust(Nimdist,method= "average") #UPGMA clustering

plot(Nimclust)

#added taxa assignment column, as determined by cluster analysis

DFA

Caret, Mass package

set.seed(3456)

trainIndex<-createDataPartition(Nimp_2t$Taxon,p=0.5,list=FALSE) # Nimp_2t$Taxon uses Taxon column to maintain proportional makeup of taxa in training/testing sets, p=0.5 performs 50/50 split in sets

data_train<-Nimp_2t[trainIndex,]

data_test<-Nimp_2t[-trainIndex,]

model<-lda(Taxon~.,data_train, prior=c(0.5,0.5)) #prior sets equal probability of a specimen being in either group to account for sample size differences

predictions<-predict(model, data_test[,-1]) # does not include taxon assignment in prediction

confusionMatrix(predictions$class, data_test$Taxon) #generates summary table and correct classification percentage

**Hoplophoneus**

Data Imputation

Norm package

Hm<-data.matrix(Hop1.1_nt) # data frame does not include taxa assignment column

s<-prelim.norm(Hm)

thetahat <- em.norm(s) #find the MLE for a starting value

rngseed(1234567) #set random number generator seed

theta <- da.norm(s,thetahat,steps=100,showits=TRUE) # take 100 steps

Himp1<-imp.norm(s,theta,Hm) #impute missing data under the MLE

Multivariate Normality

ICS package

mvnorm.kur.test(Himp)#checks for multivariate normality, essential for the following analyses

Cluster Analysis

Hopdist<-dist(Himp,method= "euclidean") #creates distance matrix for cluster analysis

Hopclust<-hclust(Hopdist,method= "average") #UPGMA clustering

plot(Hopclust)

#added taxa assignment column, as determined by cluster analysis

DFA

Caret, Mass packages

set.seed(123456)

trainIndex<-createDataPartition(Himp_3t$Taxon,p=0.5,list=FALSE) # Himp_3t$Taxon uses Taxon column to maintain proportional makeup of taxa in training/testing sets, p=0.5 performs 50/50 split in sets

data_train<-Himp_3t[trainIndex,]

data_test<-Himp_3t[-trainIndex,]

model<-lda(Taxon~.,data_train, prior=c(1,1,1)/3) #prior sets equal probability of a specimen being in either group to account for sample size differences

predictions<-predict(model, data_test[,-1]) # does not include taxon assignment in prediction

confusionMatrix(predictions$class, data_test$Taxon) #generates summary table and correct classification percentage
